# Potential Role of Vaginal Microbiota in Ovarian Cancer Carcinogenesis, Progression and Treatment

**DOI:** 10.3390/pharmaceutics15030948

**Published:** 2023-03-15

**Authors:** Xiumiao Zhao, Zhaoxia Liu, Tingtao Chen

**Affiliations:** 1 Department of Obstetrics and Gynecology, The Second Affiliated Hospital of Nanchang University, Nanchang 330006, China; 2 Queen Mary School, Nanchang University, Nanchang 330031, China; 3 National Engineering Research Center for Bioengineering Drugs and Technologies, Institute of Translational Medicine, Nanchang University, Nanchang 330031, China

**Keywords:** vaginal microbiota, ovarian cancer, probiotics, vaginal microbiota transplantation

## Abstract

Ovarian cancer represents one of the most challenging gynecologic cancers which still has numerous unknowns on the underlying pathogenesis. In addition to the verified contributors such as genomic predisposition and medical history in the carcinogenesis, emerging evidence points out the potential role of vaginal microbiota in ovarian cancer. Recent studies have underlined the presence of vaginal microbial dysbiosis in cancer cases. Increasing research also indicates the potential correlations between vaginal microbes and cancer carcinogenesis, progression and treatment. Currently, compared with other gynecologic cancers, reports on the roles of vaginal microbiota in ovarian cancer remain scarce and fragmentary. Therefore, in this review, we summarize the roles of vaginal microbiota in various gynecologic diseases, particularly focusing on the potential mechanisms and possible applications of vaginal microbiota in ovarian cancer, giving insight into the involvement of vaginal microbiota in gynecologic cancer treatment.

## 1. Introduction

Ovarian cancer is one of the most lethal gynecologic malignancies with features of high heterogeneity [1], which is estimated to occur in over 300,000 females and cause more than 200,000 deaths annually [2]. Although various therapeutic strategies including surgery, chemotherapy and maintenance therapies have been widely applied in the standard treatment [3], most cases undergo recurrence and patients suffer from a poor quality of life with the five-year survival rate lower than 50% [4,5]. Seeking a way to improve the suboptimal therapeutic effects has become an urgent task in the current research, among which the exploration on the potential elements and mechanisms involved in ovarian cancer progression might help.

Recently, with the rapid development of human microbiome projects and multi-omics analysis, the regulatory effects of vaginal microbiota in health and diseases have received significant recognition. Serving as a fibromuscular structure containing various proteins, lipids, fatty acid and glycan in the mucus [6], the vagina provides an appropriate environment for the growth of microbes. A healthy vaginal microenvironment is characterized by a *Lactobacillus*-dominated microbiota with one or several strains of *L. iners*, *L. crispatus*, *L. gasseri* or *L. jensenii* [7], which contributes to the maintenance of an acidic environment and the construction of the overall defenses to the potential pathogens [8]. However, vaginal microbial dysbiosis, generally manifesting as a deviation from the *Lactobacillus* dominated state and an increase of facultative and anaerobic organisms [9], participates in the pathogenic process of diseases such as bacterial vaginosis, preterm birth, intrauterine adhesions and infertility [10,11]. Interventions targeting the modification of vaginal microbiota have achieved great success in the cures of various diseases [12], testifying the feasibility of involving vaginal microbiota in gynecologic disease treatment.

Ovarian cancer has been found to correlate with risk factors such as genetic mutation, reproductive history and exogenous hormone use [13]; however, whether the vaginal microbes contribute to the pathogenesis has become a novel research topic. In 2019, a study reported a higher prevalence of community type O cervicovaginal microbiota with the depletion of *Lactobacillus* dominance in the presence of ovarian cancer or its related risk factors [14], which firstly connected ovarian cancer with the potential presence of vaginal dysbiosis. Among the vaginal microbiota, bacteria such as *Clostridium* and *Lachnospiraceae* are found to have positive or negative correlations with ovarian tumor development [15]. Several vaginal microbes also show anti-cancer potentials via promoting cancer cell apoptosis [16], modulating cancer-related microRNA (miRNA) expression and involvement in cancer signaling [17]. A change of the vaginal and intestinal microbiota to a specific composition induced by antibiotic treatment can inhibit the development and progression of oviductal high-grade serous carcinoma in the mouse model [15]. Based on these, there might be a non-negligible role of vaginal microbiota in the carcinogenesis, progression or even treatment of ovarian cancer.

Compared with the gut microbiota, research progress on vaginal microbiota is rather backward and scattered. Considering this, we summarize the modulatory effects of vaginal microbiota in various gynecologic diseases and explore the roles of vaginal microbiota in the carcinogenesis, progression and treatment of ovarian cancer. We also put forward the potentials of several therapeutic strategies targeting vaginal microbiota which might be used in ovarian cancer treatment. Hopefully, this evidence might help in the application of vaginal microbes in future anti-cancer treatment.

## 2. Etiology of Ovarian Cancer and the Potential Contributing Factors

Ovarian cancer comprises a collection of neoplasms with significant etiological, molecular, morphological and prognostic diversities [1], which ranks the third most common gynecologic cancer accounting for 4.7% of total cancer deaths among females [2]. The disease occurrence has been found to correlate with the state of reproductive history, exogenous hormone supplement, medical intervention, genomic predisposition and the medical history of several benign gynecologic diseases [13,18]. Serving as a heterogenous malignancy, the mechanisms involved in ovarian cancer tumorigenesis generally include the acquired or inherited defects in various genes with abnormal activation or defects in pathways such as DNA repair, mitogen-activated protein kinase (MAPK), phosphatidylinositol-3-kinase (PI3K)/Akt and the receptor tyrosine kinase (RTK)/Ras signaling pathway, the abnormal expression of hormone receptors [19] and secondary invasion from the ectopic tissues [20]. Currently, the most common treatment strategy remains the appropriate surgical staging and debulking surgery, followed by systemic chemotherapy [21]. Although radiotherapy, platinum-based chemotherapy, hormonal therapies and poly(ADP-ribose) polymerase (PARP) inhibitors maintenance therapy have also been applied in patient-specific situations [21,22], the high recurrence rate and adverse effects still hinder the achievement of expected therapeutic effects [23,24].

The exploration on the contributing factors during carcinogenesis, progression and treatment might contribute to the improvement of cancer therapy. Serving as the second genome of the human body [25], the roles of microbiota in ovarian cancer have been recognized. A study performed by Xu et al. reported gut microbial dysbiosis might be a driver of ovarian cancer via promoting epithelial–mesenchymal transition and activating tumor-associated macrophages in the cancer tissues [26]. Specific gut microbial signatures are found to correlate with the platinum sensitivity [27], and participate in the promotion of therapeutic efficacy of some antitumor drugs [28]. Meanwhile, microbes are also identified in ovarian samples with a significantly different composition between normal and cancer tissues [29]. It is known that microbiota can play roles in oncogenesis through genomic integration and genotoxicity, and have an influence on inflammation, immunity and the metabolism of the host [30]. Considering these, investigations on the change of human microbiota and their effects during cancer progression and therapies provide another viewpoint which might assist in ovarian cancer treatment.

## 3. Human Vaginal Microbiota and Its Close Relationship with Gynecologic Diseases

**Figure 1:**
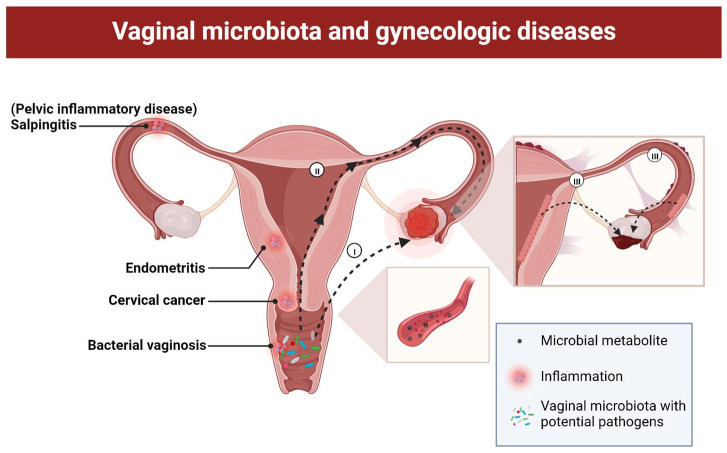
Relationship between vaginal microbiota and various gynecologic diseases and potential pathogenesis of ovarian cancer. Firstly, vaginal microbial dysbiosis has been found to correlate with the occurrence of diseases such as bacterial vaginosis, cervical cancer, endometritis and pelvic inflammatory disease. Secondly, influences of vaginal microbiota on ovarian cancer development are hypothetically achieved through (I) microbial metabolites via systemic and circulatory pathways; (II) ascension of the bacterial biofilm; (III) originating from ectopic tissues from endometrium and oviduct or secondary to benign gynecologic diseases including endometriosis and pelvic inflammatory disease. Created with BioRender.com.

The female vagina is a tubular structure lined by layers of moist stratified squamous epithelium [31], where the low pH, the presence of peroxide, organic compounds and some epithelia-derived metabolites in the vaginal fluid provide conditions for the proper growth of some specific microbes [32]. According to the bacterial communities and their relative abundances, vaginal microbiota is categorized into five clusters, with majorities referring to community state types (CST) I to III, which are dominated by species of *L. crispatus*, *L. gasseri*, *L. iners* and a few as subtypes of CST IV containing either various species of strictly anaerobic bacteria or a more bacterial vaginosis-susceptible microbiota with higher proportions of *Atopobium*, *Gardnerella*, *Prevotella* and *Parvimonas* [33]. A healthy vaginal microbiota, especially some vaginal probiotics, can help in the maintenance of the vaginal microenvironment, inhibiting potential pathogens, and regulating host immune responses [34]. Vaginal microbial dysbiosis can lead to higher susceptibility of the body to some diseases. In bacterial vaginosis, a shift of vaginal microbiota from *Lactobacillus* dominated to a polymicrobial state with an increased presence of pathogens can induce proinflammatory characteristics, immune responses and epithelium damages [32]. In addition to the localized influences on the vagina, potential correlations also exist between vaginal microbiota and diseases in the upper genital tract (Figure 1). Research on endometritis revealed the potential protective or damaging roles of vaginal microbes on the endometrium by the observation of endometritis-like symptoms after transplanting certain vaginal microbes into the rat vagina [35]. A vaginosis-associated microbiota together with *Lactobacillus* depletion, the presence of pathogens such as *Chlamydia trachomatis*, or the increase of *Atopobium vaginae* or *Porphyromonas* sp. also significantly correlate with endometriosis, pelvic inflammatory disease or even endometrial and cervical cancer [36,37,38,39,40]. It is worth mentioning that various clues also indicate the potential correlation between vaginal microbiota and ovarian cancer, which encourages a deeper exploration on the underlying mechanisms (Table 1).

## 4. Interaction between Vaginal Microbiota and Ovarian Cancer Oncogenesis and Progression

Inspired from the well-defined modulatory roles of vaginal microbiota in various gynecologic diseases, studies have turned sight to the potential interaction between vaginal microbiota and ovarian cancer. In 2019, a study reported the vaginal microbial community type O with less than 50% dominance of *Lactobacillus* spp. has a significantly higher prevalence in cases with ovarian cancer or the presence of cancer-related BRCA1 mutations [14]. In these cases, the altered types of vaginal microbiota commonly tend to be similar to those in healthy postmenopausal subjects regardless of the menopausal state, manifesting as a higher diversity of microbes and increase of *Propionibacterium* and *Corynebacterium*, even observed in the early stage I of ovarian clear cell cancer or mucinous ovarian cancer [41], which indicates that an abnormal vaginal microbiota with less protective functioning might also be implicated in ovarian cancer progression. In animal models, the development and progression of oviductal high-grade serous carcinoma can be inhibited by treating with a cocktail of metronidazole, vancomycin and streptomycin, which is regarded to correlate with microbial composition changes both in gut and vagina after antibiotic treatment [15]. Considering these, the differences of the vaginal microbiota composition between healthy and cancer patients, as well as the potential presence of microbial dysbiosis might be considered as the interfering factor in ovarian cancer progression.

Among vaginal dysbiosis, specific microbes might serve as the causative agents or the potential guardians during cancer development. The serological findings of antibodies against *C. trachomatis*, such as Pgp3 and CHSP60-1, are found to associate with an increase of ovarian cancer risk, which might be mediated by the promotive effects of the pathogens on the survival of DNA-damaged host cells or the transfer of tubal derived cells to the growth-promoting microenvironment in the ovaries [42,43]. On the contrary, a negative correlation between *Clostridium* XIVa species in both fecal and vaginal microbiome and ovarian cancer tumor scores has been reported, which is hypothesized to result from the high butyrate productivity of the microbes, since the researcher stated that despite its promotive effects on cancer progression via the induction of regulatory T cells (Tregs), the existed inhibitory effects might outweigh in this process as butyrate can also interfere with ovarian cancer cell growth in vitro [15]. Moreover, with the increasing recognition on the role of inflammation in ovarian cancer carcinogenesis [51], microbes might participate in the tumor progression through the induction of inflammation-related signaling pathways. Kelly et al. demonstrated that the bacterial products, for example, the lipopolysaccharide isolated from *Escherichia coli*, can induce the production of pro-inflammatory cytokines of ovarian tumor cells, promote tumor growth and the development of chemo-resistance to Paclitaxel potentially through the TLR-4-MyD88 signaling pathway [44]. The presence of a toll-like receptor (TLR) and its related signaling in tumor cells can participate in the promotion of an inflammatory response and cell survival in various cancer types, thus the identification of TLR on ovarian cells and its potential mechanisms suggesting that microbes might also interfere with ovarian cancer progression through promoting TLR signaling [52,53]. Furthermore, in our recent study on the intratumor microbes in epithelial ovarian cancer, microbes have also been testified to promote cancer progression potentially through the activation of an inflammation-induced hedgehog signaling pathway [45].

The mechanisms on the establishment of the relatively long-distance connection between vaginal microbiota and the progression of ovarian cancer remains ambiguous. As shown in Figure 1, in addition to the potential of systemic and circulatory pathways [54], the microbiota continuum and structural continuity along the female genital tract might provide another theoretical basis [55]. It has been already testified in the pathogenesis of other gynecologic disease that, in the exploration on the microbial characterization of endometrial cancer, apart from the microbial signature change in the cancer cases, a high concordance of microbiota between all organs in the genital tract ranging from the vagina to the ovaries has also been observed [38]. It is revealed that bacteria can ascend from the vagina to the upper genital tract in bacterial vaginosis [56], and there is a microbial continuum along the female genital tract on account of a wide existence of facultative anaerobes and aerobes observed in the reproductive organs [57]. Therefore, the distant influences of vaginal microbiota on the upper genital tract might be mediated by the upward movement or transfer of the vaginal microbes [38]. On the other hand, in terms of ovarian cancer, vaginal microbiota might serve as the indirect risk factor in carcinogenesis, as the cancer can sometimes occur secondarily to several gynecological diseases and some of the subtypes have been widely accepted to ectopically originate from the fallopian tubes or endometrium [19]. Previous studies have revealed that the presence of pelvic inflammatory disease or endometriosis might predispose the occurrence of ovarian cancer, which is potentially led by the ectopic tissue origin of ovarian cancer from extraovarian epithelial cells or genomic instability triggered by a sustained inflammatory microenvironment [37,58,59].

## 5. Interaction between Vaginal Microbiota and Ovarian Cancer Treatment

### 5.1. Surgery

Normally, the fluctuation of vaginal microbial composition is tightly associated with the estrogen level and menstrual cycle, manifesting as a significant shift in the period of menopause [60,61]. Since a deficiency of circulating estrogen in menopause can lead to changes in the urogenital tissues such as a reduced glycogen and collagen content and thinning of the epithelium, an alteration of vaginal microbiota and loss of *Lactobacilli* dominance are frequently observed [62,63]. In ovarian cancer treatment, invasive procedures such as debulking cytoreductive surgery and hysterectomy will inevitably cover oophorectomy in the high-grade malignancies, inducing postmenopause-like symptoms such as vaginal and sexual dysfunction, premature menopause and osteoporosis due to the change of hormone and its regulation [64]. Compared with that of postmenopausal women, oophorectomy causes a lack of estradiol and testosterone and an absolute loss of estrogen secretion, predisposing a vaginal status in patients similar to those who are healthy postmenopausal [65,66]. In animal models, it leads to a lower total bacterial load and absence of *Lactobacillus*, accompanied by an increase of vaginal pathogens including *Clostridium perfringens*, *Bacteroides*, *Staphylococcus epidermidis* and *S. aureus* [46]. Meanwhile, this altered microbiota also correlates with the vaginal symptoms. The oophorectomy-related symptoms such as vaginal atrophy is accompanied by increased bacterial diversity with decreased *Lactobacillus* species and the downregulation of genes for the maintenance of the epithelial structure and barrier function [67]. The significant compositional change is hypothesized to be primarily led by the effects of estrogen on *Lactobacillus* spp., as the vaginal glycogen level, which serves as the preferred substrate for *Lactobacilli,* is regarded to be modulated by estrogen secretion [46,60].

### 5.2. Chemotherapy

Since anti-cancer drugs are widely distributed to non-targeted sites, systemic chemotherapy always leads to severe adverse effects in cancer treatment. During this process, significant changes of microbiota have been identified and there might be complicated interactions between the symptoms and microbes. In the chemotherapy-induced intestinal mucositis, gut microbial dysbiosis and the related functional disorders contribute to abnormal metabolism and signal transduction in the gut [68], which might interfere with the pathogenesis by modifying the inflammatory environment, integrity of the intestinal barrier, innate immunity and repair function [69]. Meanwhile, the break of the epithelial barrier further lead to the formation of a pseudomembrane on the ulceration and translocation of gut microbes [70]. Chemotherapy has been found to cause elevated levels of inflammatory cytokines in the plasma [71], and alter adaptive immunity via depleting circulating lymphocytes [72]. Therefore, widely distributed inflammatory reactions and changes of immunity might make the body more susceptible to pathogen infections. In the vagina, changes of inflammation and immunity are found to correlate with bacterial vaginosis and vulvovaginal Candidiasis [73]. Considering these, chemotherapy-induced vaginal dysbiosis might be unavoidable in the cancer treatment.

On the other hand, increasing evidence has revealed the potential correlations between microbiota and the achievement of therapeutic efficacy during ovarian cancer chemotherapy. In the gut microbiota, specific signatures of dysbiosis manifesting as a remarkable decrease of diversity and increase of instability have been identified in platinum-resistance patients, indicating that microbial dynamics might serve as the therapeutic target for ovarian cancer [27]. Similarly, in more than 30% of the patients with primary platinum-resistance ovarian cancer, an alteration of vaginal microbiota dominance turning from *Lactobacillus* to *Escherichia* has also been observed [47]. Although the exact mechanism of action is still obscure, Jacobson et al. pointed out that the shortened platinum-free interval is possibly attributed to the impacts of *Escherichia* on ROS production or its facilitation on immune response and cancer growth [47]. Moreover, the study also revealed an association between *L. iners* and lowered gross residual disease, suggesting that a potential microbial characteristic in the vagina might serve as a biomarker for the therapeutic efficacy [47].

### 5.3. Radiotherapy

Radiotherapy can sometimes serve as the adjuvant or consolidative treatment following or combining surgery and chemotherapy in ovarian cancer, which might cover from the focused site of the tumor to the whole pelvic or abdomen [22,74]. In this intervention, systemic side effects frequently occur, especially in the use of wide-field irradiation [74]. For example, radiotherapy can lead to intestinal epithelial damage and dysfunction of the epithelial barrier, which is found to be strongly correlated with the dysbiosis of gut microbiota [75]. In terms of the effects on the vagina, symptoms including vaginal stenosis, dyspareunia, insufficient vaginal lubrication and changes of the vaginal structure including the epithelial and mucosal atrophy as well as reduced epithelium volume are also observed [76,77]. Moreover, increasing evidence has revealed the influences of radiotherapy on vaginal microbiota. Compared with the women who are undergoing the pre-radiation therapy stage or healthy postmenopausal, radiotherapy significantly increases the abundances of species which are typically rare in the healthy vaginal microbiota, including the dysbiosis of several members of the *Lachnospiraceae* family, *Mobiluncus*, *Atopobium* and *Prevotella* [48,49]. At the same time, the depletion of *Lactobacillus* spp. is also observed and these altogether contribute to the susceptibility of bacterial vaginosis after radiotherapy [48]. To be more precise, a study led by Tsementzi et al. specifically revealed the association between vaginal symptoms and individual bacterial taxa after radiotherapy, such as the correlations between *Prevotella* and vaginal dryness, and *Gemillaceaea* and reduced lubrication during sexual intercourse, which further indicate an unstable vaginal microbiota caused by cancer radiotherapy [50]. Radiotherapy can damage the vaginal epithelium, leading to inflammation and local cell death [78]. In the meantime, the existence of vaginal pathogens as well as the higher-diversity microbiota also lead to the high mucosal inflammation level by elevating the concentrations of inflammatory cytokines [79].

### 5.4. Other Treatments Which Might Interact with Vaginal Microbiota

In recent years, the modulatory effects of gut microbiota on anti-cancer therapy have been well developed [80]. Studies on immune checkpoint blockade therapy targeting the PD-1/PD-L1 axis showed that performing fecal microbiota transplantation from immune checkpoint inhibitor reactive cancer patients improves the efficacy of the programmed cell death protein 1 (PD-1) blockade [81,82]. FMT leads to a positive change of immune cell infiltration and gene expression in the gut lamina propria and tumor microenvironment, among which, enteric bacteria including *Akkermansia muciniphila* and *Bifidobacterium* play a prominent immune regulatory role [81,82,83]. Although a more specific mechanism remains to be studied, the utilization of microbes or microbiota in cancer immunotherapy has greatly inspired people. The possible immunoregulatory effects of microbes are hypothetically achieved through its stimulatory effect of microbial antigens on the T cell response, the engagement of pattern recognition receptors and the release of microbial metabolites [84]. Among these, researchers have put forward the idea on involving toll-like receptors in ovarian cancer immunotherapy, as TLR activation might contribute to an anti-tumor response in immune cells despite its promotive roles on the tumor cell itself [85]. In an examination of vaginal microbiota composition and immune checkpoint proteins, a positive correlation between TLR2 levels in the cervicovaginal microenvironment and vaginal *Lactobacillus* dominance is observed [86]. Considering that the vaginal probiotics *L. crispatus* can mediate the differentiation of monocytes to Langerhans-like cells probably via activating the TLR2/6-TFs-CD207 axis in an in vitro experiment [87], the study inferred that *L. crispatus* might participate in the regulation of antitumor immunity [86]. Programmed death ligand 1 (PD-L1) and lymphocyte activation gene-3 (LAG-3) are also found to negatively correlate with and be dependent on the vaginal *Lactobacillus* dominance; these altogether indicate the potential immune regulatory effect of vaginal microbiota during carcinogenesis [86].

## 6. Targeting Vaginal Microbiota in Ovarian Cancer

Taking advantages of the local and systemic influences of gut microbiota on shaping immunity and the treatment-related response and toxicity, various therapeutic strategies based on modulating gut microbiota in cancer therapy have been well investigated and achieved great success in the correction of microbial disorders [88]. According to the research findings in the previous section, one can anticipate that strategies on manipulating vaginal microbiota in order to reconstitute the normal composition or taking advantage of special characteristics of specific bacterial strains might also be involved in ovarian cancer treatment for the achievement of adjuvant therapeutic effects. Here, the roles of potential bacterial strains and their applications in disease treatment, as well as several conventional and novel therapeutic strategies, are altogether discussed as follows.

### 6.1. Antibiotics

Antibiotics have been involved in the treatment of gynecological infections such as bacterial vaginosis and sexually transmitted diseases with the aim of eliminating pathogens and reestablishing vaginal eubiosis [89]. As microbial dysbiosis and potential pathogens are widely identified in gynecological diseases, the correction of microbiota with antibiotics has provided another viewpoint in disease intervention. In endometriosis and cervical cancer, the occurrence or disease progression can be significantly inhibited by the antibiotic treatment either by removing the related pathogens or altering the microbial richness and diversity [90,91]. Furthermore, the antibiotic cocktail that reduced advanced tumor progression in a mouse model features as the formation of two special clusters of vaginal and fecal microbiota after antibiotic treatment, one of which correlates with fewer and less advanced tumors compared with the control microbiota composition [15]. Several antibiotics such as minocycline, erythromycins and tetracyclines have also been found to have a direct bearing on cancer cells [54], although the in vivo effects remain to be explored.

Nevertheless, antibiotics can also pose negative effects in the process of cancer treatment. In the recurrent endometrial, cervical and ovarian cancer cases, pretreatment with antibiotics leads to decreased response and survival rates in the following immunotherapy [92]. Similarly, in the explorations targeting gut microbiota, researchers pointed out the development of cisplatin resistance and accelerated tumor growth after antibiotic treatment in epithelial ovarian cancer was associated with disruption of the gut microbiota [93]. So far, there has not been a study testifying the actual effects and underlying mechanisms on the antibiotic-induced vaginal microbial change in ovarian cancer; however, the aspects mentioned above might provide the potential cut-in point for future research. Additionally, antibiotic treatment can lead to unwanted adverse effects such as an excess risk of infection in ovarian cancer cases [94], which should be carefully assessed and considered before application.

### 6.2. Single-Probiotic Supplement and Probiotic Combination Strategies

**Figure 2:**
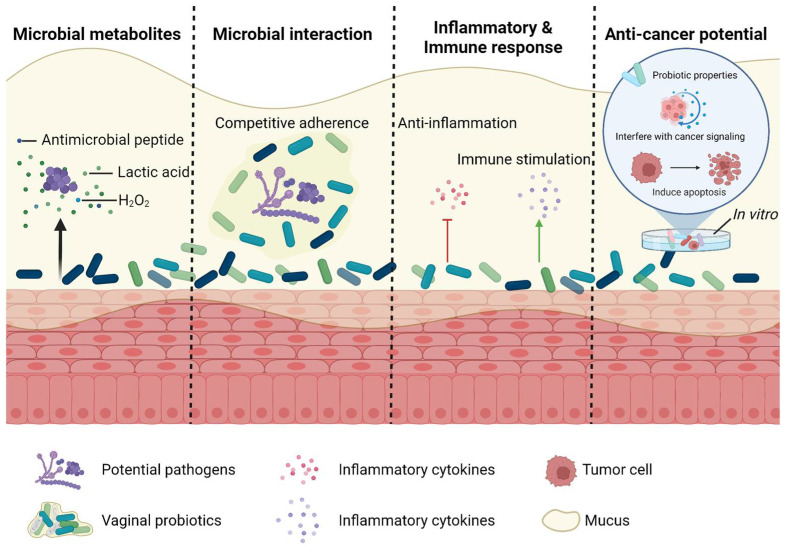
Potential roles of vaginal probiotics in ovarian cancer adjuvant therapy. Firstly, vaginal probiotics can produce defense factors to maintain a general vaginal environment. Secondly, vaginal probiotics can interact with potential pathogens and rescue microbial dysbiosis. Thirdly, vaginal probiotics can regulate the host inflammatory and immune response. Fourthly, in the in vitro experiment, some bacterial strains manifest probiotic properties and anti-cancer potentials, which might pose apoptotic effects on cancer cells or interfere with the cancer signaling. Created with BioRender.com.

Probiotics can help maintain or restore the balance of microbiota, thus being regarded effective in the treatment of increasing gynecological diseases. For example, a live biotherapeutic product Lactin-V composed of a natural vaginal strain of *L. crispatus* has been originally testified as useful in preventing the recurrence of bacterial vaginosis and applied in clinical studies [95]. It is found to combat microbial dysbiosis via reconstituting the vaginal colonization of *L. crispatus* [96], and sustainedly reducing an abundance of bacterial vaginosis-associated genera such as *Prevotella* spp. and *Megasphaera* spp. [97]. Additionally, modulatory roles on genital inflammation and the biomarkers of epithelial integrity have been further identified, leading to the hypothesis on its potential influences on HIV susceptibility [97]. Apart from the single-strain preparations, probiotic combinations have shown great potentials in rescuing dysbiosis, as the therapeutic effects might be enhanced by the potential synergy and additive effects of the individual components [98]. Our previous study has revealed that vaginal interventions via a probiotic combination of five selected *Lactobacillus* strains can successfully rescue vaginal dysbiosis in rats [99]. In the clinical trial, the use of a probiotic mixture of *L. acidophilus* GLA-14 and *L. rhamnosus* HN001 in combination with lactoferrin significantly improved the recurrence rate of bacterial vaginosis and the Nugent score [100]. Vaginal probiotics is known to maintain a lower pH under anaerobic conditions, provide lactate by-product with anti-microbial activity and produce hydrogen peroxide to inhibit potential pathogens [101], contributing to the precondition of a healthy vaginal environment. Vaginal dysbiosis is a widespread manifestation in various gynecological disorders [9]. Therefore, although there have been few studies involving vaginal microbiota in ovarian cancer treatment, it can be inferred that several mechanisms found in specific probiotic and microbial strains might also participate in the modulation and improvement of the therapeutic process (Figure 2).

Interaction with the potential pathogens and rescuing microbial dysbiosis. It is known that vaginal probiotics, especially *Lactobacillus* spp., can produce various defense factors such as lactic acid, hydrogen peroxide and bacteriocins to maintain a general environment which is not suitable for the growth and colonization of pathogens [102]. The vagina is estimated to contain the highest putative bacteriocin genes compared with other body sites [103], among which, *Lactobacillus* spp. seems to play leading roles as bacteriocin genes widely and massively found in their genome [103]; meanwhile, some of their products manifest a broad-spectrum antimicrobial capacity towards multiple pathogens [104]. Based on these, *Lactobacillus* spp. might help in building up an overall antimicrobial defense system in the vagina. In addition, the direct interaction between vaginal microbiota and pathogens can be achieved through their competitive adherence. In vitro experiments have shown that several vaginal *Lactobacillus* strains can interfere with the adhesion of *Listeria monocytogenes*, *Streptococcus agalactiae*, *S. aureus* and *Gardnerella vaginalis* on vaginal epithelial cells, as *Lactobacilli* might occupy or mask the binding sites of the mucosal to exclude the colonization of pathogens, compete for the receptor sites to adhere on the epithelial surface and displace the pathogens [105,106,107]. In clinical studies, the vaginal administration of a *Lactobacilli* combination after standard treatment can increase the therapeutic efficacy and prevent relapse in vaginal *Candida albicans* infections, which is supposed to be mediated by the protective roles of *Lactobacilli* and its competitive adhesion on the epithelium [108]. Additionally, the association of two *Lactobacillus* stains *L. fermentum* and *L. plantarum* tend to provide a long-term protection towards pathogens and significantly reduces the Nugent score and restores the physiological vaginal microbiota in the cases with bacterial vaginosis [109].

Regulations on inflammatory and immune response. Genital inflammation is crucial in fighting infection and recruiting the host immune response, while elevated mucosal inflammatory cytokine levels can associate with altered mucosal function, impaired epithelial barrier integrity and induced HIV susceptibility [110]. It is generally regarded that the *Lactobacillus*-dominated vaginal microbiota is associated with an overall low inflammation with low levels of proinflammatory cytokines such as tumor necrosis factor alpha (TNF-α) and high levels of anti-inflammatory or regulatory cytokines such as interleukin-1 receptor antagonist (IL-1Ra) and interleukin-10 (IL-10) [79]. Therefore, vaginal probiotics might contribute to the regulation of the inflammatory state and immune response in the vaginal environment. In *C. albicans* infections, the local administration of *L. crispatus* in animal models maintains immune homeostasis by decreasing the release of proinflammatory cytokines and potentially inducing antibody-mediated protection through elevated epithelial-derived immunoglobulin G (IgG) expression [111]. Furthermore, several other *Lactobacillus* strains or probiotic combinations also show in vitro potentials in decreasing the pathogens- or TLR-induced elevated pro-inflammatory cytokine production [112,113]. This balances the T helper 1/T helper 2 (Th1/Th2) ratio and inhibits nuclear factor-κB (NF-κB) signaling in the vaginal epithelium, and alleviates the potential damage of the epithelial barrier and tissue injury caused by a sustained elevated cytokine release [110,111,112,113]. In humans, the administration of *Lactobacilli* vaginal tablets significantly reduces interleukin-1β (IL-1β) and (interleukin-6) IL-6 secretion in bacterial vaginosis, testifying the modulatory effects of *Lactobacilli* on the vaginal inflammatory response [114]. On the other hand, specific strains also contribute to the increase of specific inflammatory levels, suggesting that *Lactobacilli* also participate in the promotion of host defense [115]. Apart from the inhibitory roles on the expression of NF-κB-related inflammatory genes, the combination of *L. rhamnosus* GR-1 and *L. reuteri* RC-14 elevates interleukin-α (IL-1α) and IL-1β levels, indicating that *Lactobacilli* might play modulatory roles on the inflammatory response by activating an alternate signal transduction pathway [113].

Anti-cancer roles of special vaginal microbes. With the deeper exploration on the characteristics of vaginal microbes, direct anticancer potentials have been found in specific bacterial strains, which might contribute to the modulation of ovarian cancer treatment. By separately culturing cancer and normal cell lines with microbial metabolites isolated from *L. acidophilus* 36YL, *L. plantarum* MTCC 9510, *L. plantarum* 5BL and *Enterococcus faecalis*, expected probiotic properties and apoptotic effects on cancer cells without cytotoxicity on normal cells are observed [16,116,117,118]. In the ovarian cancer CAOV-4 cell line, vaginal-isolated *Lactococcus lactis* exhibits a comprehensive influence including downregulations on TLR-4, miR-21 and miR-200b expression levels, which have been regarded to correlate with ovarian cancer initiation, metastasis, recurrence and the buildup of chemo-resistance [17]. Although the underlying mechanisms remain to be evaluated, these altogether provide a prospect for the topical use of vaginal probiotics and applications of microbial anticancer compounds in cancer treatment.

### 6.3. Vaginal Microbiota Transplantation

In recent years, a tendency to regulate microbiota integrally instead of singly targeting on specific pathogenic strains has become a focal point in disease intervention. Among these, fecal microbiota transplantation (FMT), referring to transferring fecal microbial content from a healthy donor into the gut of patients, has significant therapeutic effects in diseases such as *Clostridioides difficile* infection, inflammatory bowel disease, irritable bowel syndrome and autism [119]. In these diseases, FMT might function through directly competing with the intestinal pathogens, stimulating mucosal immunity, targeting on the microbiota–gut–brain axis, and most commonly and significantly, rescuing dysbiosis of the gut microbiota [120,121]. FMT has also been testified as effective in the alleviation of ovariectomy-induced vaginal atrophy in animal models, which takes an indirect effect on the recovery of vaginal tissues through modulating gut microbiota [122]. Moreover, the discovery on the role of FMT in immunotherapy has raised the awareness of applying microbiota transplantation in cancer therapy [123].

**Figure 3:**
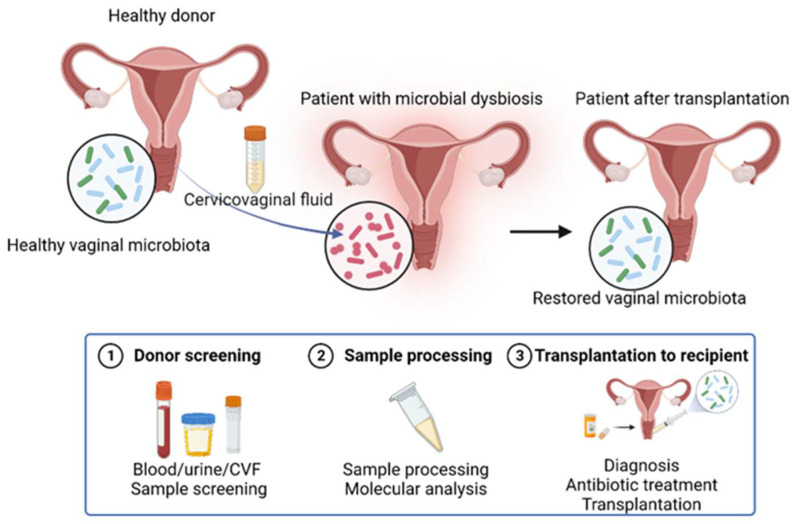
Schematic depiction of VMT. Firstly, donors are recruited and screened including analysis on blood, cervicovaginal fluid (CVF) and urine samples. Secondly, CVF samples are collected from donors and processed. Thirdly, CVF with healthy vaginal microbiota is transplanted into the vagina of recipients to restore the normal vaginal microbiota and rescue the normal function. Created with BioRender.com.

Considering the similar characteristics of the gastrointestinal tract and the vagina, such as the secretory function and bacterial colonization on the epithelium, the dynamic alterations of the microbiota in the state of homeostasis and dysbiosis, as well as the potential relationship between the specific microbes and diseases, vaginal microbiota transplantation (VMT) has also become a novel research topic in these recent years (Figure 3).

Studies on VMT first started with exploring the therapeutic effects on correcting vaginal dysbiosis and achieved positive outcomes. In 2019, Lev-Sagie et al. performed VMT on five patients suffering from severe bacterial vaginosis, and four of them obtained long-term alleviation, manifesting as a relief of symptoms and recovery of a *Lactobacillus*-dominated vaginal microbiota without significant adverse effects [124]. Our study further revealed that VMT can achieve similar therapeutic effects with a probiotic combination on the correction of vaginal dysbiosis in rats [99]. Additionally, evidence has also pointed out the potentials of VMT in other gynecological diseases. Recently, we reported that VMT might be a novel treatment strategy for endometriosis or other diseases, as there are modulatory effects on the NF-κB signaling pathway, the expression of inflammatory cytokines, cell proliferation marker and macrophage marker observed in the endometrial lesion in the mouse model [90]. Furthermore, the regulatory effects of VMT on reducing the infiltration of immune cells were also observed in the uterine wall while correcting vaginal dysbiosis [99]. Therefore, more comprehensive regulatory effects of VMT might be achieved as it retains the complete capacity of the healthy vaginal microbiota.

On account of the great potentials of VMT, here we hypothesize that VMT might also be applied in the management of ovarian cancer therapy and the related complications with the feasibility discussed as follows: (i) Vaginal dysbiosis is also found during ovarian cancer progression and widely exists as a complication after cancer treatment. (ii) Specific vaginal microbes in the healthy vagina might participate in the regulation of ovarian cancer progression and treatment. (iii) The potential undefined mechanisms underlying the host–microbe interaction as well as the function of vaginal microbiota as a whole from the healthy individuals might help in ovarian cancer treatment. As a result, the specific and direct effects of VMT on ovarian cancer remains to be testified either for the cancer treatment itself or for the alleviation on the complications. Furthermore, from the perspective of VMT, the construction of a standardized process and criteria, as well as the solutions to related ethical issues might promote the VMT from the experimental stage to clinical applications.

## 7. Conclusions and Future Directions

Ovarian cancer is one of the most severe gynecological cancers with a relatively low survival rate. Although novel treatments and improved strategies have been explored, the therapeutic effects are still unsatisfactory and various complications frequently occur. Recently, applications of gut microbiota in anti-cancer therapy have been well studied and achieved great success. Similar to the gastrointestinal tract, the vagina also contains microbiota with great potential in the treatment of gynecological diseases. It is found that vaginal dysbiosis and abnormal microbes also exist in the pathogenesis and progression of ovarian cancer and widely serve as the complications of anti-cancer therapy. In this review, we summarize the potential roles of vaginal microbiota in the management of ovarian cancer therapy both in the treatment and in the relief of their related complications. We also put forward potential strategies such as probiotic supplements and vaginal microbiota transplantation of modulating vaginal microbiota in ovarian cancer therapy. So far, there is no integral research on the modulatory effects of vaginal microbiota on ovarian cancer treatment and some mechanisms are not well understood. Due to the great potential, it is necessary to further explore the therapeutic effects of modulating vaginal microbiota as well as taking advantages of vaginal probiotics in ovarian cancer treatment.

## Figures and Tables

**Table 1:** Ovarian cancer-related alteration of microbiota and potential interactions.

Intervention	Study Subject	Microbial Signature	Potential Interactions	Reference
Observation in cancer cases	Human body	Identified community with less than 50% dominance of *Lactobacillus* spp. (vaginal microbial community type O) in cancer cases	Microbial community type O has higher prevalence in ovarian cancer and cases with BRCA1 mutation	Nené et al. [14]
	Human body	Higher microbial diversity in cancer cases Increase of *Propionibacterium* and *Corynebacterium* Similar to those in healthy postmenopausal subjects	Ovarian cancer correlates with microbiome alteration at the site distant from the tumor tissue	Morikawa et al. [41]
	Human body	*C. trachomatis* infection correlates with an increase of ovarian cancer risk	Prolonged infection results in The facilitation of DNA-damaged cells survival, which may promote cancer initiation	Trabert et al. Ness et al. [42,43]
	Cancer cells in vitro	Lipopolysaccharide isolated from *Escherichia coli* can promote the production of proinflammatory cytokines and tumor survival	Bacterial products promote cancer progression potentially through the activation of TLR-4-MyD88 signaling pathway	Kelly et al. [44]
	Human body	Intratumor bacteria *Propionibacterium acnes* increase as a key strain in cancer progression	Intratumor bacteria promote cancer progression potentially through the activation of hedgehog (Hh) signalling pathway	Huang et al. [45]
	Cancer cells in vitro	*Lactococcus lactis* downregulates the expression of TLR-4, miR-21 and miR-200b in CAOV-4 cells	Vaginal isolated probiotics present great potentials on the control of ovarian cancer tumorigenesis, metastasis, recurrence and overall survival rate	Rahbar Saadat et al. [17]
Antibiotic treatment	Animal models	Antibiotic-induced alteration of microbiota composition Specific microbial signature positively or negatively correlates with the tumor scores	Microbiota composition influences ovarian cancer development and progression	Chen et al. [15]
Surgery	Animal models	Lower total bacterial load Absence of *Lactobacillus* and anaerobic bacteria Increase of *Clostridium perfringens*, *Bacteroides*, *Staphylococcus epidermidis* and *Staphylococcus aureus*	Dysbiosis caused by absence of ovarian hormones	Bezirtzoglou et al. [46]
Chemotherapy	Human body	Loss of *Lactobacillus* dominance >20% relative abundance of *Escherichia*	Microbial signature correlate with platinum resistance and ovarian cancer prognosis	Jacobson et al. [47]
Radiotherapy	Human body	Increase of *Lachnospiraceae* family, *Mobiluncus*, *Atopobium* and *Prevotella* Lower abundances of *Lactobacillus*, *Gardnerella* and *Peptostreptococcus*	Therapy-induced microbial dysbiosis	Bai et al. [48]
Radiotherapy	Human body	Higher α-diversity Higher abundance of typically rare species	Treatment-induced changes of abundance of key species which produce mucopolysaccharides	Tsementzi et al. [49]
Radiotherapy	Human body	Identified correlation between specific vaginal symptoms and individual bacterial taxa	Therapy-induced reduced microbiome stability and symptom persistence	Tsementzi et al. [50]

## Data Availability

Not applicable.
